# Hello handsome! Male's facial attractiveness gives rise to female's fairness bias in Ultimatum Game scenarios—An ERP study

**DOI:** 10.1371/journal.pone.0180459

**Published:** 2017-07-05

**Authors:** Qingguo Ma, Da Qian, Linfeng Hu, Lei Wang

**Affiliations:** 1 School of Management, Zhejiang University, Hangzhou, China; 2 Neuromanagement Lab, Zhejiang University, Hangzhou, China; Macquarie University, AUSTRALIA

## Abstract

The current study delineated how male proposers’ facial attractiveness affect female responders’ fairness considerations and their subsequent decision outcome during the Ultimatum Game (UG). Event Related Potentials (ERPs) were recorded from 17 female subjects, who played the role as responders and had to decide whether to accept offers from either attractive or unattractive male proposers. Behavioral data (Acceptance Ratio and Response time) revealed that, more offers were accepted from attractive-face conditions; subjects typically responded quicker to unfair offers from unattractive proposers as compared with slower to unfair offers from attractive proposers. The ERP data demonstrated similar N2 amplitudes elicited by both attractive and unattractive faces, and a larger early frontal LPP elicited by the attractive faces compared with unattractive ones, but no significant differences of both late posterior LPP and typical parietal LPP amplitudes were observed between these two face conditions, which was different from our previous study with similar paradigm but male participants. The results suggest that, in comparison to males, females might not experience the potential attention bias towards unattractive opposite-sex faces and are less likely to possess an enhanced processing and evaluation of those faces. This phenomenon might be explained by endogenous gender differences in mate preference. The feedback-related negativity (FRN) and P300 responses during an offer presentation were further measured in both attractive-face and unattractive-face conditions and the results demonstrated that the amplitudes elicited by fair and unfair offers were not statistically different in the former condition, but were different in the latter condition. More specifically, unfair offers generated larger FRN and smaller P300 than fair ones in the unattractive-face condition. Findings suggest that, although females tend to possess less salient evaluation of male’s facial attractiveness, the attractiveness of male proposers would still attenuate female responders’ fairness consideration during the UG.

## 1. Introduction

As a classical example of economic games, the Ultimatum Game (UG) developed by Güth and colleagues [1], is frequently adopted to investigate how individuals make strategic decisions during social interactions. This game also enables scholars to study how people respond to offers on the basis of different fairness levels. In a typical two person UG, the responder can either accept or reject the allocator’s offer depending on the responder’s perception of fairness. Responders will accept the offer if it is deemed as fair enough, and then both the responder and the allocator will split the pie as proposed; however, both of them will receive nothing if the offer is turned down by the responder, and the game between this allocator and this responder ends so that reciprocation is not an issue. Of course, the game can be performed continuously between two other persons. Abundant studies demonstrated that responders in the UG would often consider rejecting unfair or unsatisfying offers that violate the so-called social norm of fairness [2–4]. In studies that explored the effects of emotions on decision making, unfair offers would trigger negative emotions toward the allocator, and in turn, lead to punishment of such socially unexpected behavior [5]. It was further evidenced in the research of Fehr and Gächter [6] that most people are well aware that they trigger strong negative emotions when they free ride, which would subsequently lead to punishment from others. Hence, allocators in the UG would consider giving an appropriate offer to avoid gaining nothing from the game. In addition, an array of social factors such as social distance, initial ownership of asset, income contribution and female’s facial attractiveness were found in studies to play an influential role on people’s fairness consideration during economic games [7–11]. Take social distance for example, Wu et al [11] adopted a dictator game to explore the effect of social distance on people’s fairness evaluation. They observed different expectancy of social norm violation between friends and strangers, and in turn, suggested that social distance could modulate the evaluation of this violation.

It is widely acknowledged that a highly attractive individual would capture our attention easily and influence how we construe him/her [12]. Langlois and colleagues [13] argued that we have a greater tendency to be biased towards attractive individuals, because they are perceived as being physically healthier [14], or to have increased fertility [15] or possess stronger socializing skills and have better job performance [16, 17], hence they tend to be well paid and have greater mating success [18]. In the ultimatum game conducted by Solnick and Schweitzer [4], it was noted that proposers were willing to offer more money to attractive responders than to unattractive responders. Moreover, responders accepted more unfair offers from attractive proposers than from unattractive proposers, indicating that the notion of “beauty premium” was evidenced in which attractive people were treated differently by others. However, some other existing literatures challenged the simple “Beauty Premium Hypothesis”. Krupp and colleagues [19] tested whether this effect is due to attractiveness *per se* by experimentally manipulating apparent health, which is a reliable component of attractiveness, in a Trust Game (TG) and found that participants did not invest more in but return more to attractive partners, indicating a preference for reciprocation with those attractive partners. This was also supported with a finding from another study that attractiveness facilitated trusting behavior only when participants knew that their partners could see their faces [20]. These findings suggest that when contacting with attractive persons, participants are expecting their reciprocation rather than simply feeling rewarded from attractiveness [19, 20]. Furthermore, an intriguing gender difference was observed in Krupp et al [19]’s study that participants fairly reciprocated the trust of healthy more than unhealthy female players, but not of healthy versus unhealthy male players. That is to say, being attractive or not for females would influence others’ reciprocation of their trust. The more attractive those females are, the more returns they will receive. However, being attractive for males doesn't necessarily provide this benefit [19]. This result is consistent with our previous behavioral findings in the “female proposer, male responder” UG [7], such that unfair offers proposed by attractive females were accepted more often than similar offers from unattractive females, but whether it will still be applicable in “male proposer, female responder” UG remains in question. On the other hand, an interesting recent study by Fugère and colleagues [21] found that women and their mothers were strongly influenced by the physical attractiveness and preferred the attractive target men, and later expressed higher dating desirability. Men with the most desirable personality profiles were rated more favorably than their counterparts only when they were at least moderately attractive. Unattractive men were never rated as more desirable partners for daughters, even when they possessed the most desirable trait profiles [21]. Therefore, it predicts that males’ physical attractiveness is an indispensable base for females’ mate preference judgement. Taking together from the study of Solnick and Schweitzer [4] mentioned above, that participants offered more to attractive males, even though attractive males did not demand more. Therefore, a finding that attractive males may also influence female’s fairness consideration and subsequent choice would well complement the above studies.

An extensive body of neurophysiological researches with non-human primates [22, 23] or functional Magnetic Resonance Imaging (fMRI) studies with humans [24–26] has demonstrated the monetary equivalent role of facial attractiveness, and also indicated the temporal stages of neural processing toward facial attractiveness as well as the spatial display of how specific brain areas were involved in processing that reward kind, and its subsequent action. In a recent ERP study, Jin and colleagues [27] investigated how borrowers’ facial attractiveness influenced lenders attitudes toward repayment behavior of those borrowers. They found that attractive faces (of borrowers) induced smaller early negativity (regarded as N2, typically peaking around 200–300 ms post-onset face stimuli) than unattractive faces, and interpreted this finding as attention bias towards opposite sex-faces due to male’s desire to view attractive faces [27], which is consistent with our previous work [7, 28]. Chen et al [29] found N2 and late positive potential (LPP) associated with the processing of attractive and unattractive faces, this LPP also regarded as late positive component (LPC), often occurs at central-parietal scalp sites and peaks between 300–700 ms post-onset face stimuli [30]. It is found to be more positive for attractive than unattractive or even neutral faces and has been interpreted as related to engagement towards motivationally significant stimuli [31–33]. More specifically, facial recognition studies show that typical LPP are commonly divided into an earlier frontal LPP in 300–500 ms and a later more posterior LPP in 500-700ms time range [30, 34], and found attractive faces trigger larger early frontal LPP than unattractive faces [35]. In the experiment conducted by Zeng and colleagues [36], subjects had to decide whether to view attractive female pictures or obtain monetary benefit while their brain activities were recorded. The results illustrated that, compared to monetary benefits, subjects’ choices to view attractive females generated a larger late positive component during 450–650 ms time window, thus the authors concluded that viewing attractive female faces are more direct and emotionally rewarding for males than money [36]. In another ERP study, Ma et al [7] explored the temporal features of facial attractiveness processing, and discovered that attractive faces would elicit a smaller early negativity (N2) and a larger LPP in comparison to unattractive faces, reinforcing the views of Zeng et al [36] that there exist a “beauty premium effect” during social interactions [7]. To sum up, all the above studies suggested that participants could distinguish between attractive and unattractive faces in the neuro level, based on the ERPs observed during face presentation. Attractive females might be perceived as socially rewarding, and facial attractiveness would play an equivalent role as other monetary rewards. We would expect the underlying neural mechanisms to be similar in this study.

It has been documented in a number of ERP studies that brain responses to different offers during the UG is generally indicated by the feedback-related negativity (FRN) and P300 [11, 37–39], which are considered as the two major ERP components well-established to differentiate one’s reaction to positive and negative outcomes. FRN, a negative reflection of ERP, is often understood as the distinctive brain responses towards positive feedback (such as task success, obtain reward or avoid punishment) and negative feedback (such as task failure, non-reward or punishment after task). It peaks approximately between 200 and 350 ms after outcome presentation, and it is generated from the anterior cingulate cortex (ACC) and nearby cortical areas, especially in the medial-frontal region of the ACC [40, 41]. FRN was first reported by Miltner et al [42] that it represents the neural substrates after an incorrect feedback. It also reflects the neural processing of gains or losses as an enhanced negative amplitude was elicited in a loss scenario than it was generated in a gain scenario [40]. Reinforcement learning theory (RL-theory) originally developed and proposed by Holroyd and Coles [43] extended the error monitoring hypothesis by Miltner et al [42] and suggested that the increased ACC activity was due to the decreased phasic activation in the midbrain when the outcome was worse than expected, and in turn, resulted in a more pronounced FRN amplitudes [44]. In the light of the above RL-theory, many existing studies found that unfair offers elicited a more negative-going FRN compared to fair offers and these results suggested that, the FRN reflects a rapid impression as to whether outcomes violate social norms [37, 39, 45]. Therefore, similar to these studies, in the current experiment, we would expect to observe a larger FRN waveforms elicited by the unfair offers. Furthermore, in our previous experiment using the “female allocator, male responder” UG, we found different FRN results across attractive and unattractive face conditions. In the former condition, the FRN responses to either fair or unfair offers were not statistically different. Interestingly, in the latter condition, an enlarged FRN was elicited by unfair offers [7]. This intriguing inconsistency suggested that one’s facial attractiveness would potentially modulate others’ fairness considerations, and this was in line with preceding literatures which state that the condition of the allocator could promote diverse brain responses relative to various fairness levels [9, 11]. Therefore we predict the similar FRN effect would follow in this study.

In addition to FRN, P300 represents stimuli evaluation after the feedback presentation, and it is a positive ERP component with the most positive peak between 300 and 600 ms at the centro-parietal recording sites, after feedback is presented [46]. P300 amplitudes normally increase from frontal to parietal sites and it is understood as representing higher-order cognitive operations such as decision-making [47], or attentional resource allocation [48]. Yeung and Sanfey [46] asserted that P300 component is closely related to the subjects’ evaluation of reward magnitude and valence, with larger reward or positive feedback during outcome stage associated with a more positive P300 amplitude [44, 49, 50]. Wu et al [8] extended our understanding of game theory by manipulating subjects’ feeling of initial ownership in the UG. In the task of how to divide 10 yuan between the allocator and responder, the authors observed that participants, who acted as responders, perceived that the fairest offer for themselves was about 6.5 yuan when they initially owned that 10 yuan. However, their fairest offer expectation was significantly reduced to 5 yuan when the whole amount was initially owned by the allocator. Following that, the authors found that the existence of an enlarged P300 during fair offers compared with unfair offers. In our study, we expect to observe the similar pattern of P300.

Previous behavioral experiments investigated the effects of facial attractiveness on fairness considerations and indicated that people may be both more generous to and demanding of attractive people [4, 51]. Other studies have suggested that in comparison of females, males are more motivated by good-looks of the opposite sex to discount larger future monetary rewards in exchange of fewer immediate monetary rewards [52, 53]. However, very few studies have discussed the temporal dynamics of this process, and most of those studies are mainly using female as proposers in the UG [7, 27, 28] to explore male responders’ choices induced by fairness consideration. An interesting fMRI study [53] though, found Orbital Frontal Cortex (OFC) activation only for men during an attractiveness judgement task, and highlighted this pattern might be due to potential gender differences in judgment criteria towards facial attractiveness. The authors predicted that men may have prominent sexual appeal in their judgement of female faces whereas women may have just followed a predetermined aesthetic judgement of male faces, suggesting that the “beauty premium” might have more salient effect on males [53]. While the above study tested the gender differences towards facial attractiveness judgement using a higher spatial resolution fMRI, the present study was designed to investigate (using a higher temporal resolution ERPs) whether the judgement of attractive and unattractive proposers in a modified UG would alter interaction partners’ fairness consideration, in the context of male proposers with female responders. That is, will the “beauty premium” effect of males still exist and alleviate female responders’ fairness consideration? Or more specifically, will female responders’ FRN waveforms be attenuated due to alleviated fairness consideration toward attractive male allocators? Moreover, in the work of Shackelford and colleagues [54], they proposed a universal dimension of human mate preferences and identified several component of attractiveness (eg. Intelligence, health, good looks etc.) which would likely affect perception of attractiveness and subsequent behavior such as reciprocation noted by Krupp et al [19] above. Since we adopt a typical two player UG, reciprocation is not an issue, nevertheless, it would be interesting to test for a relationship between facial attractiveness and offer acceptance rate in the UG after controlling for individual variation. That is, is there still an effect of facial attractiveness on the acceptance ratio when individual differences are controlled for? In order to answer these questions, we adopted a similar experimental design from our previous experiment [7] by presenting 180 photographs of male allocators (half attractive, half unattractive) to 18 female students recruited, and at the end of experiment, we asked them to complete a follow up questionnaire to measure individual differences toward components of attractiveness. The findings will extend our understanding on identifying sex differences in the neural substrate underlying the processing of facial attractiveness.

## 2. Material and methods

### 2.1 Participants

A total of eighteen healthy, right-handed, heterosexual female subjects aged between 20–25 years (Mean = 22.08 years, SE = 1.58 years) were randomly selected from the campus of Zhejiang University to participate in this experiment. They were all native Chinese undergraduates, had normal or corrected-to-normal vision and self-reported that they didn’t have any brain injury, mental disease or neurological disorders in the past. All subjects were scanned at the Neuromanagement Lab of Zhejiang University. The internal review board and the Ethical department of Neuromanagement lab approved this experiment. Before the formal experiment was initiated, written informed consent were collected from all participants. One subject's ERP data had excessive recording artifacts so it was removed, and we were left with 17 valid subjects for the final analysis eventually.

### 2.2 Experimental procedure

Face stimuli consisted of a set of unfamiliar unnamable faces to the subjects (i.e. no celebrities) and were mainly gathered from online face database and internet. Photoshop software were adopted to gray process faces and edit them to a standard (4.5 by 4 cm, 220 by 200 pixels) size. Forty female students from other universities rated a total of three hundred Chinese male photos for their attractiveness using a 7 point scale (1 being “not attractive at all” and 7 being “extremely attractive”) before the ERP experiment was carried. We then sorted the orders (from highest to lowest scores) based on the ratings, and selected photos from top 90 faces and bottom 90 faces to form two groups, namely attractive-face group (Mean = 5.99, SD = 0.27, Range = 1.17, from 5.63 to 6.8) and unattractive-face group (Mean = 2.77, SD = 0.15, Range = 0.81, from 2.18 to 2.99). Independent sample t-test was conducted to examine whether the ratings were significantly different (t_(178)_ = 100.2, p < 0.001, Cohen’s *d* = 14.93) between the two groups. The result indicated significant difference in terms of attractiveness.

Placed in a dim, sound-attenuated, electrically shielded room, all female subjects were seated comfortably in front of a 17-in. CRT display (which is used to present the experimental stimuli in the center of the screen) at 100cm away, with a visual angle of 8.69° × 6.52° (15.2 × 11.4 cm, width × height). Subjects were instructed to read and understand the experimental task rules and were encouraged to ask questions before the formal experiment, subjects were also assigned 10 practice trials to familiarize themselves with the procedure. A number keypad was provided to the subjects to make choices (press 1 for acceptance, 3 for rejection). The whole experiment involved three blocks, each block had 60 trials (half of those trails contain attractive male faces and the other half contain unattractive male faces). There were a total of five offer conditions (with unfair offers 1:9, 2:8; mid-value offer 3:7; fair offers 4:6, and 5:5), and each condition was repeated 36 times. More specifically, under the attractive face condition (90 faces), unfair offers 1:9 and 2:8 were repeated 18 times respectively, and were added up together (36 times) as unfair offer condition; fair offers 5:5 and 4:6 were each repeated 18 times, and were added up together (36 times) as fair offer condition; mid-offers 3:7 were repeated 18 times. It was the same distribution for the unattractive face condition. Offers were randomly assigned within each face set so that fair and unfair offers would not be biased toward higher or lower rated faces of each face set. In addition, each male photo will appear only once throughout the whole experiment to rule out the effect of repetition.

**Fig 1:**
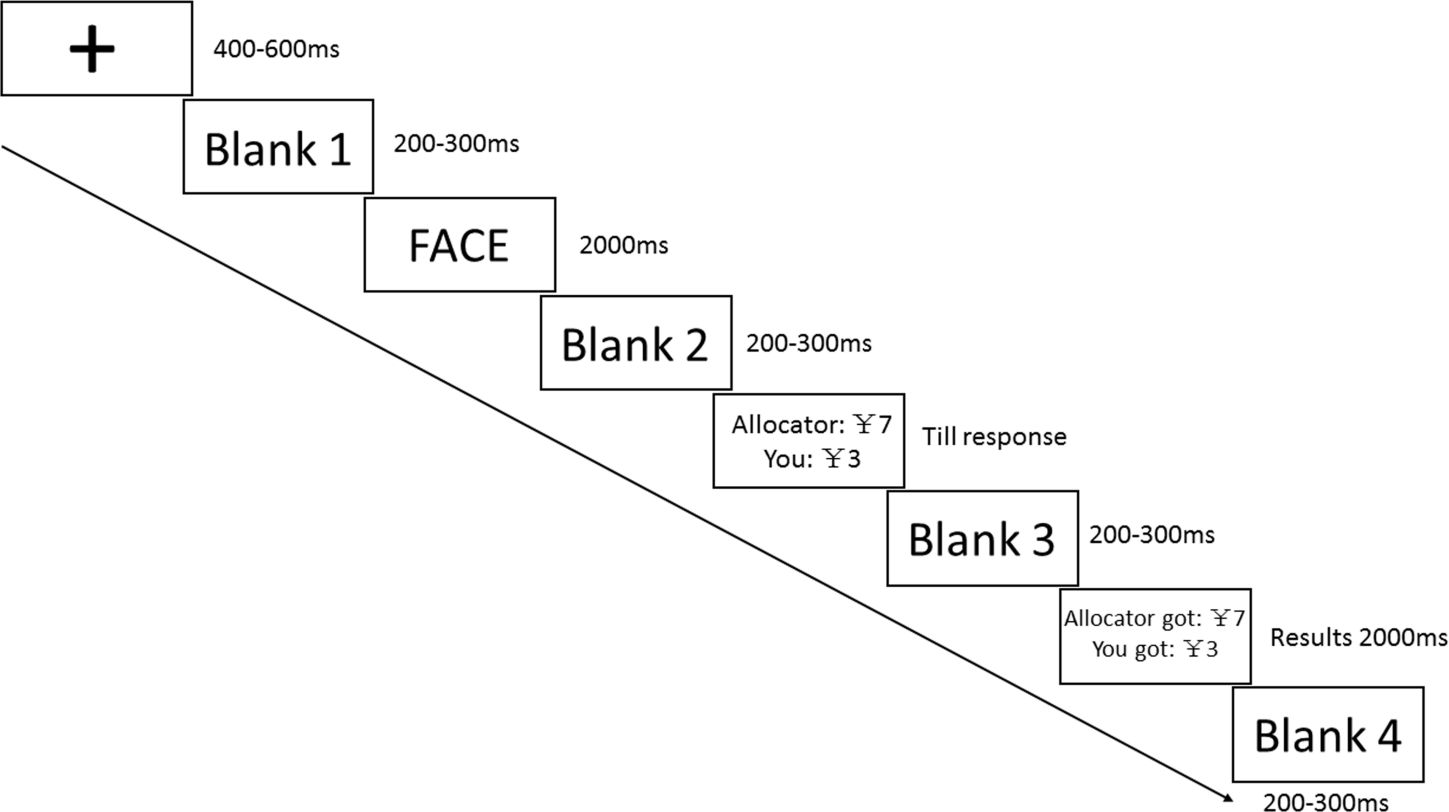
A single trial of the experimental procedure.

As depicted in Fig 1, for each trial, a fixation first appeared for 400–600 ms, then followed by a blank screen for 200–300 ms, and after that, the face of allocator (either attractive or unattractive) was presented for 2000 ms followed by another blank screen lasting about 200–300 ms. The screen then displayed the allocator’s offer to split Ұ10. There was no time limit for the subject (as responder in the game) to make a decision by pressing the number keypad. If the offer is rejected, both the allocator and the subjects will be empty handed; otherwise, they will split the money as proposed by the allocator. Once the responder made the decision, another blank screen appeared for 200–300 ms, and then the screen subsequently showed the final money distribution of this trial for 2000 ms, this was followed by another blank screen (200–300 ms) before continuing to another trial. Each Subject earned Ұ35 (approximately $5.38) for her participation, and was informed that based on her decision results, one of the trails will be randomly selected as a bonus towards their final payment. Also we asked subjects whether or not they would allow us to take their photos after the experiment, and the photos would be used for future experiments upon their agreement. We used E-prime 2.0 software package (Psychology Software tools, Pittsburgh, USA) to operationalize the presentation of stimuli, recording trigger and button. In addition, since we were also concerned about whether facial attractiveness would indeed affect participants’ choices in this study, follow up questionnaires were administered upon completion of the experiment to explore how important facial attractiveness is to them (using 5-point scale, 5 being extremely important, 1 being not important at all) when judging males’ attractiveness in general, along with ratings of other popular factors namely social status, resource, achievement, intelligence and healthy body. We then sorted the orders (of importance towards males’ attractiveness) of the above six factors and applied paired t-tests to compare the differences among them.

### 2.3 EEG recordings

We recorded subjects’ EEGs (band-pass 0.05–70 Hz, sampling rate 500 Hz) using a 64 scalp sites electro cap and the Neuroscan Synamp2 Amplifier (Neurosoft Labs, Inc.). We chose the left mastoid as online reference and the average of the left and right mastoids were served as off-line reference for the EEGs. The ground electrode was connected to the forehead location. We then recorded Vertical electrooculograms (EOGs) with one pair of electrodes placed in parallel above and below the left eye and recorded horizontal EOGs by placing the other pair of electrodes at the orbital rim of both left and right eyes. During the experiment, the electrode impedance was maintained less than 5 KΩ.

### 2.4 Data analysis

For the behavioral data analysis, there were five different offers (fair offers: 5:5, 4:6; Mid-value offer: 3:7; unfair offers: 2:8, 1:9) from two types of proposers (attractive and unattractive), so the data went into a two × five factorial design. Therefore it resulted in a total of 10 conditions for the repeated measure ANOVA analysis to compare the acceptance ratios (ARs) and response times (RTs). We used the Greenhouse-Geisser correction for sphericity departures and a post-hoc analysis using the Bonferroni correction was followed afterwards.

For the pre-processing of EEG data, Neuroscan 4.3.1 (Neurosoft Labs, Inc.) was adopted. Correction method proposed by Semlitsch et al [55] was adopted to correct EOGs artifacts with ocular movements. The data were then digitally filtered through a zero-phase shift below 30 Hz (24 dB/Octave). We treated the pre-stimulus period as the baseline while segmented EEG recordings for the epoch from the time window of 200 ms before the stimuli onset to 800 ms post-onset. We visually inspected each set of the EEG raw data for potential artifacts, and trials which were affected by amplifier clipping, bursts of electromyographic activity, or peak-to-peak deflection exceeding ± 80μV were discarded from further averaging analysis. A total of two conditions (attractive-face and unattractive-face) were resulted after the averaging of the EEG epochs for the attractive (mean trails = 85, minimum trails = 81) and unattractive faces (mean trails = 84, minimum trails = 82) during the photo presentation, and a total of four conditions: attractive-fair (mean trails = 34, minimum trails = 32), attractive-unfair (mean trails = 33, minimum trails = 31), unattractive-fair (mean trails = 34, minimum trails = 31), and unattractive-unfair (mean trails = 33, minimum trails = 32) were resulted after the averaging of Attractiveness (attractive/unattractive face) and Offer (fair/unfair offers) respectively during the offer presentation.

**Fig 2:**
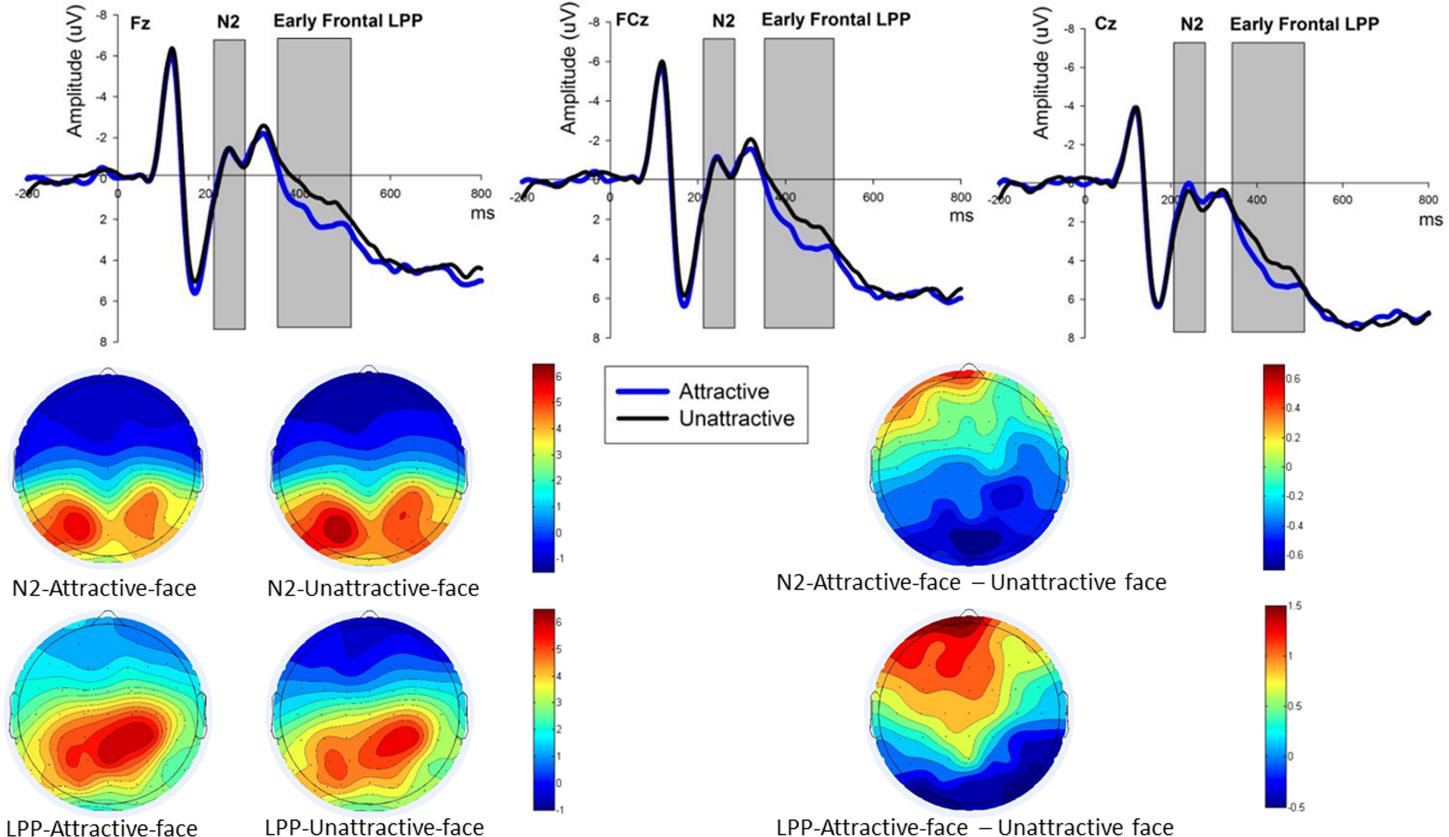
The ERP grand-average waveforms and topographic maps of N2 and early frontal LPP. (A) Fz, FCz and Cz were selected for N2 (shaded 210–280 ms time window) and LPP (shaded 380–480 ms) waveforms to compare among “attractive”, “unattractive” and “attractive minus unattractive” face conditions. (B) The bars for attractive and unattractive faces of the N2, early frontal LPP, N2 difference, LPP difference topographic maps range from -1.5μV to 6.5μV, -1μV to 6.5μV, -0.7μV to 0.7μV and -0.5μV to 1.5μV respectively.

As our previous study [7], we considered the N2 and LPP during face processing. Based on the visual inspection and scalp distribution (see Fig 2), we chose nine frontal-central electrodes (F3, Fz, F4, FC3, FCz, FC4, C3, Cz and C4) that were the same as Ma et al [7] to analyze the averaged amplitudes of N2 in the 210–280 ms post-onset of face presentation. Considering that the face might be either attractive or unattractive, the data went into a two (attractiveness) × nine (electrodes) repeated measure ANOVA to observe the neural processing of the allocators’ facial attractiveness. The scalp map of LPP (see Fig 2) also showed a pronounced typical parietal LPP amplitudes in the central-parietal area as our previous study [7], therefore six electrodes (CP3, CPz, CP4, P3, Pz, P4) which were the same as Ma et al [7] were chosen for the analysis of typical LPP (from 300–700 ms) post-onset the face presentation so the data then went into a two (attractiveness) × six (electrodes) repeated measure ANOVA. However, according to the scalp map of difference between attractive and unattractive conditions (see Fig 2), this difference mainly distributed in the frontal-central region. Therefore, nine electrodes (F3, Fz, F4, FC3, FCz, FC4, C3, Cz and C4) were selected for early frontal LPP (380–480 ms) analysis [30, 35]. Correspondingly, a late posterior LPP (500–700 ms) post-onset the face presentation at the six electrodes (CP3, CPz, CP4, P3, Pz, P4) was analyzed. As our previous study [7], we considered the FRN and P300 during the outcome processing. Based on the scalp distribution which indicated that more obvious FRN response were observed at frontal sites, data from the same nine electrodes (F3, Fz, F4, FC3, FCz, FC4, C3, Cz and C4) in Ma et al [7] were analyzed, and was followed by the FRN amplitude averaging process from the time range 260–320 ms post-offer presentation. Since our primary concern in this study was to compare the FRN and P300 effects in fair (offers 5:5, 4:6) and unfair (offers: 1:9, 2:8) conditions, mid-value offers (3:7) were excluded from both FRN and P300 analysis. FRN data went into a two (attractiveness: attractive/unattractive) × two (offer: fair/unfair) × nine (electrodes) repeated measure ANOVA post-offer presentation. Also given that the maximum P300 amplitudes were observed at parietal sites, data from six electrodes (CP3, CPz, CP4, P3, Pz and P4) as in Ma et al [7] were analyzed in the 460–560 ms time range post-offer presentation, while a two (attractiveness: attractive/unattractive) × two (offer: fair/unfair) × six (electrodes) repeated measure ANOVA was applied. If the interaction effect between Attractiveness and Offer was significant, a simple effect analysis was followed. When necessary, we applied the Greenhouse-Geisser correction and Bonferroni correction in all of our statistical analysis.

To further address the abovementioned research question that the “Beauty Premium” effect would whether or not exist in the female participants’ paradigm, we retrieved the male participants’ data from previous work [7] to form a new sample (total of 38 participants involving 21 male participants from previous study and 17 female participants from this study) and adopted a two (attractiveness) x nine (electrodes) mixed model ANOVA (using participants’ sex treated as between-subjects factor) to inspect gender effects on face processing. It is worth noting that the male data for N2 and early frontal LPP were captured in the same time window (210- 280ms for N2 and 380-480ms for early frontal LPP, respectively) and electrode sites (F3, Fz, F4, FC3, FCz, FC4, C3, Cz and C4) for better comparability. If the interaction effect between Attractiveness and Gender was significant, a simple effect analysis was followed, and Greenhouse-Geisser correction and Bonferroni correction were applied in the statistical analysis.

## 3. Results

### 3.1 Acceptance ratios

During the two (attractive vs. unattractive) × five (fair offers: 5:5, 4:6; Mid-value offer: 3:7; unfair offers: 2:8, 1:9) ANOVA analysis, main effects of Attractiveness [F_(1,16)_ = 13.192, p = 0.002, partial η^2^ = 0.452] and Offers [F_(4,64)_ = 31.532, p < 0.001, ε = 0.463, partial η^2^ = 0.663] were identified. Specifically, the average acceptance ratio in attractive-face condition was significantly higher than that in unattractive-face condition (Mean = 67.1%, SE = 5.4%; Mean = 51%, SE = 7.1%, respectively). The post-hoc comparisons showed that acceptance ratio of fair offer (5:5) was significantly higher than unfair offers (1:9) (t_(16)_ = 7.172, p < 0.001, Cohen’s *d* = 1.74), (2:8) (t_(16)_ = 6.319, p < 0.001, Cohen’s *d* = 1.53), and mid-value offer (3:7) (t_(16)_ = 4.910, p = 0.002, Cohen’s *d* = 1.14), but there was no significant difference (t_(16)_ = 2.048, p = 0.545, Cohen’s *d* = 0.50) observed between fair offers (4:6) and (5:5). A comparison between unfair offers (1:9) and (2:8) was not significant (t_(16)_ = 2.571, p = 0.198, Cohen’s *d* = 0.61). Mid-value offer (3:7) was accepted more than unfair offer (1:9) (t_(16)_ = 3.538, p = 0.026, Cohen’s *d* = 0.86). The interaction effect between Attractiveness and Offer was also significant [F_(4,64)_ = 4.358, p = 0.004, partial η^2^ = 0.214], and the simple effect analysis indicated that attractive faces triggered higher acceptance rates across all five offer (1:9; 2:8; 3:7; 4:6; 5:5) conditions compared with corresponding offers proposed by unattractive-faces, as depicted in Table 1. Moreover, the post-hoc comparisons indicated that, in the attractive face condition, acceptance rates for fair offers (5:5) and (4:6) were not different (t_(16)_ = 2.13, p = 0.437, Cohen’s *d* = 0.52), but they were significantly higher than the rest of offers (offer 5:5 vs offer 3:7, t_(16)_ = 3.99, p = 0.011, Cohen’s *d* = 0.97; offer 5:5 vs offer 2:8, t_(16)_ = 5.79, p < 0.001, Cohen’s *d* = 1.40; offer 5:5 vs offer 1:9, t_(16)_ = 7.13, p < 0.001, Cohen’s *d* = 1.73; offer 4:6 vs offer 3:7, t_(16)_ = 3.94, p = 0.012, Cohen’s *d* = 0.96; offer 4:6 vs offer 2:8, t_(16)_ = 5.76, p < 0.001, Cohen’s *d* = 1.40; offer 4:6 vs offer 1:9, t_(16)_ = 7.09, p < 0.001, Cohen’s *d* = 1.72). Offer (3:7) was different with offer (2:8) (t_(16)_ = 3.41, p = 0.037, Cohen’s *d* = 0.83) and offer (1:9) (t_(16)_ = 4.26, p = 0.006, Cohen’s *d* = 1.03). Unfair offers (2:8) and (1:9) were not different from each other (t_(16)_ = 2.62, p = 0.175, Cohen’s *d* = 0.64). A similar pattern was observed in the unattractive face condition, except offer (3:7) was not different with unfair offers (2:8) (t_(16)_ = 1.73, p > 0.9, Cohen’s *d* = 0.42) and (1:9) (t_(16)_ = 2.27, p = 0.382, Cohen’s *d* = 0.55).

**Table 1:** The acceptance ratio (ARs) and response time (RTs) across two face conditions during the offer presentations.

OFFERS	(5:5)	(4:6)	(3:7)	(2:8)	(1:9)
ARs(%)	99.6±0.4	96.5±1.4	64.2±0.9	42.4±9.9	32.6±9.5
ARs (%)	89.8±4.5	75.6±7.9	37.9±10.4	28.2±9.7	23.4±9.7
SIG	0.038	0.007	0.001	0.009	0.034
ATTRACTIVE FACE (HIGHLIGHTED) VS. UNATTRACTIVE FACE					
RTs(ms)	899.97±94.92	1235.02±165.04	1516.29±189.15	1472.88±156.68	1097.10±116.84
RTs (ms)	1062.98±124.26	1366.75±165.44	1471.44±152.32	1296.46±135.01	959.68±87.43
SIG	0.031	0.226	0.705	0.061	0.036
ATTRACTIVE FACE (HIGHLIGHTED) VS. UNATTRACTIVE FACE					

### 3.2 Response times

Two (attractive vs. unattractive) × five (fair offers: 5:5, 4:6; Mid-value offer: 3:7; unfair offers: 2:8, 1:9) ANOVA results of response time indicated that there was an insignificant difference [F_(1,16)_ = 0.117, p = 0.737, partial η^2^ = 0.007] between RTs of taking an action when viewing Attractive and Unattractive faces, however, main effect of offer [F_(4,64)_ = 16.098, p < 0.001, ε = 0.704, partial η^2^ = 0.502] was significant during ANOVA analysis. The post-hoc comparisons revealed that subjects responded the quickest to fair offer (5:5) (Mean ± SE, 981.476 ± 105.098), which turned out to be a more rapid decision making than fair offer (4:6) (t_(16)_ = 4.064, p = 0.009, Cohen’s *d* = 0.99), mid-value offer (3:7) (t_(16)_ = 6.112, p < 0.001, Cohen’s *d* = 1.48) and unfair offer (2:8) (t_(16)_ = 5.583, p < 0.001, Cohen’s *d* = 1.35), but not for unfair offer (1:9) (t_(16)_ = 1.675, p > 0.9, Cohen’s *d* = 0.41). A significant interaction effect between Attractiveness and Offer [F_(4,64)_ = 2.845, p = 0.031, partial η^2^ = 0.151] also existed, and the follow-up simple effect analysis revealed that RTs were shorter for fair offer (5:5) and longer for unfair offer (1:9) when offers were made by attractive faces in comparison to the unattractive ones (depicted in Table 1). The RTs were not different for other pairs of offers. Furthermore, in the attractive-face condition, we observed significant differences in RTs between fair offer (5:5) with unfair offers (2:8) (t_(16)_ = 5.337, p = 0.001, Cohen’s *d* = 1.29) and (1:9) (t_(16)_ = 3.304, p = 0.045, Cohen’s *d* = 0.80), however, in the unattractive-face condition fair offers (5:5) were not different with those unfair offers (2:8) (t_(16)_ = 2.426, p = 0.274, Cohen’s *d* = 0.59) and (1:9) (t_(16)_ = 1.481, p > 0.9, Cohen’s *d* = 0.34). In both attractive and unattractive face-conditions, mid-value offers (3:7) were significantly different with the two extremely fair (5:5) (for attractive: t_(16)_ = 5.072, p = 0.001, Cohen’s *d* = 1.23; for unattractive: t_(16)_ = 3.709, p = 0.019, Cohen’s *d* = 0.90) and unfair (1:9) (for attractive: t_(16)_ = 3.552, p = 0.027, Cohen’s *d* = 0.86; for unattractive: t_(16)_ = 5.321, p = 0.001, Cohen’s *d* = 1.29) offers respectively, but there were no significant differences between the RTs of mid-value offer (3:7) and the RTs of relative fair (4:6) (for attractive: t_(16)_ = 2.471, p = 0.251, Cohen’s *d* = 0.60; for unattractive: t_(16)_ = 0.949, p > 0.9, Cohen’s *d* = 0.23) and unfair (2:8) (for attractive: t_(16)_ = 0.383, p > 0.9, Cohen’s *d* = 0.09; for unattractive: t_(16)_ = 2.011, p = 0.615, Cohen’s *d* = 0.49) offers.

### 3.3 N2 and LPP

A two (Attractiveness) × nine (Electrodes) ANOVA analysis for N2 and early frontal LPP were carried respectively. We identified insignificant main effects of Attractiveness [F_(1,16)_ = 0.190, p = 0.669, partial η^2^ = 0.012] for N2 amplitudes (210–280 ms post-onset the face stimuli) in the frontal-central electrodes with no statistical differences of amplitude among any pairs of electrodes between the attractive-face (Mean ± SE, 0.888 ± 0.838 μV) and the unattractive-face conditions (Mean ± SE, 0.772 ± 0.889 μV). A significant early frontal LPP (380–480 ms post-onset the face stimuli) effect [F_(1,16)_ = 10.858, p = 0.005, partial η^2^ = 0.404] was identified when comparing attractive faces (Mean ± SE, 2.382 ± 1.152 µV) against less attractive ones (mean ± SE, 1.205 ± 1.084 µV), with more pronounced early frontal LPP elicited by attractive faces. A two (attractiveness) × six (electrodes) ANOVA analysis for late posterior LPP (500–700 ms post-onset face stimuli) and typical LPP (300–700 ms post-onset face stimuli) identified insignificant main effects of attractiveness for late posterior LPP [F_(1,16)_ = 1.069, p = 0.317, partial η^2^ = 0.063] and typical LPP [F_(1,16)_ = 0.193, p = 0.667, partial η^2^ = 0.012]. Interaction effect between Attractiveness and Electrode was not significant for N2 [F_(8,128)_ = 1.246, p = 0.301, ε = 0.476, partial η^2^ = 0.072], early frontal LPP [F_(8,128)_ = 2.343, p = 0.072, ε = 0.449, partial η^2^ = 0.128], late posterior LPP [F_(5,80)_ = 1.656, p = 0.197, ε = 0.515, partial η^2^ = 0.094] and Typical parietal LPP [F_(5,80)_ = 2.169, p = 0.112, ε = 0.538, partial η^2^ = 0.119] respectively. (Fig 2 illustrates the insignificant N2 and significant early frontal LPP waveforms).

### 3.4 FRN

**Fig 3:**
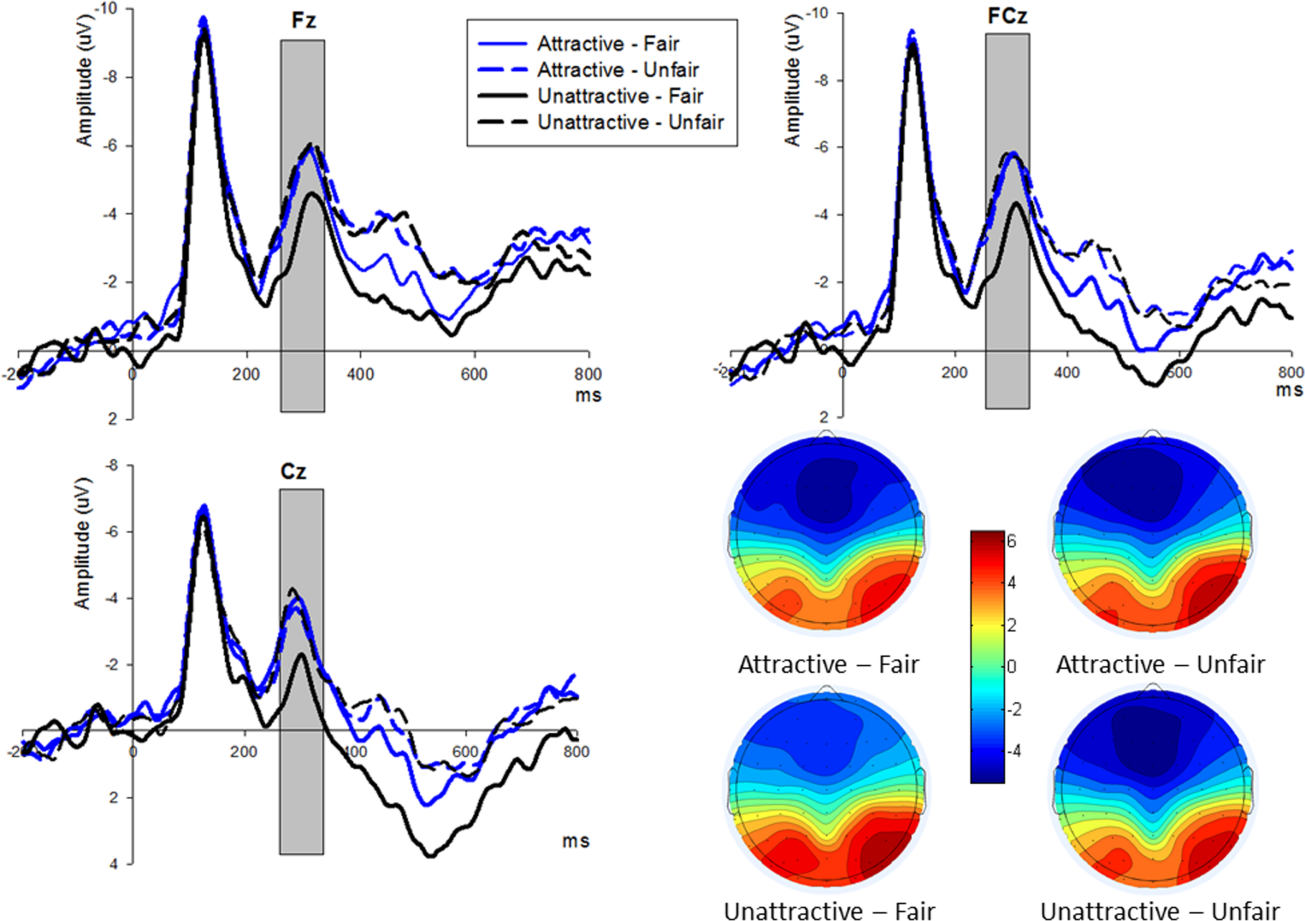
The ERP grand-average waveforms and topographic map of FRN. (A) Fz, FCz, and Cz were selected for FRN (shaded 260–320 ms) waveforms to compare fair and unfair offers in the two face conditions. (B) The bar for four offer conditions of FRN topographic map ranges from -5.5μV to 6μV.

We identified significant effects of Attractiveness [F_(1,16)_ = 5.922, p = 0.027, partial η^2^ = 0.270] and Offer [F_(1,16)_ = 4.786, p = 0.044, partial η^2^ = 0.230] when we conducted a two (Attractiveness) × two (offers) × nine (Electrodes) ANOVA analysis for the FRN (260–320 ms post-onset the offer presentation), As illustrated above, offer (5:5) and offer (4:6) were treated as one offer condition (fair offers), whereas offer (1:9) and offer (2:8) were treated as another offer condition (unfair offers). We have excluded the mid-value offers (3:7) from analysis as mentioned in the methods section. The FRN waveforms and topographic map are illustrated in Fig 3. In addition, the interaction effect (between Attractiveness and Offer) also approached significance [F_(1,16)_ = 4.403, p = 0.052, partial η^2^ = 0.216]. In the attractive-face condition [F_(1,16)_ = 0.008, p = 0.931, partial η^2^ < 0.001], similar FRN responses were generated by the unfair offers (Mean ± SE, −3.556 ± 0.761 μV) compared with the fair ones (Mean ± SE, −3.604 ± 0.884 μV). In the contrary, unfair offers (Mean ± SE, −3.709 ± 0.620 μV) elicited more negative amplitudes [F_(1,16)_ = 8.925, p = 0.009, partial η^2^ = 0.358] than fair ones (Mean ± SE, −2.023 ± 0.797 μV) in the unattractive-face condition.

### 3.5 P300

**Fig 4:**
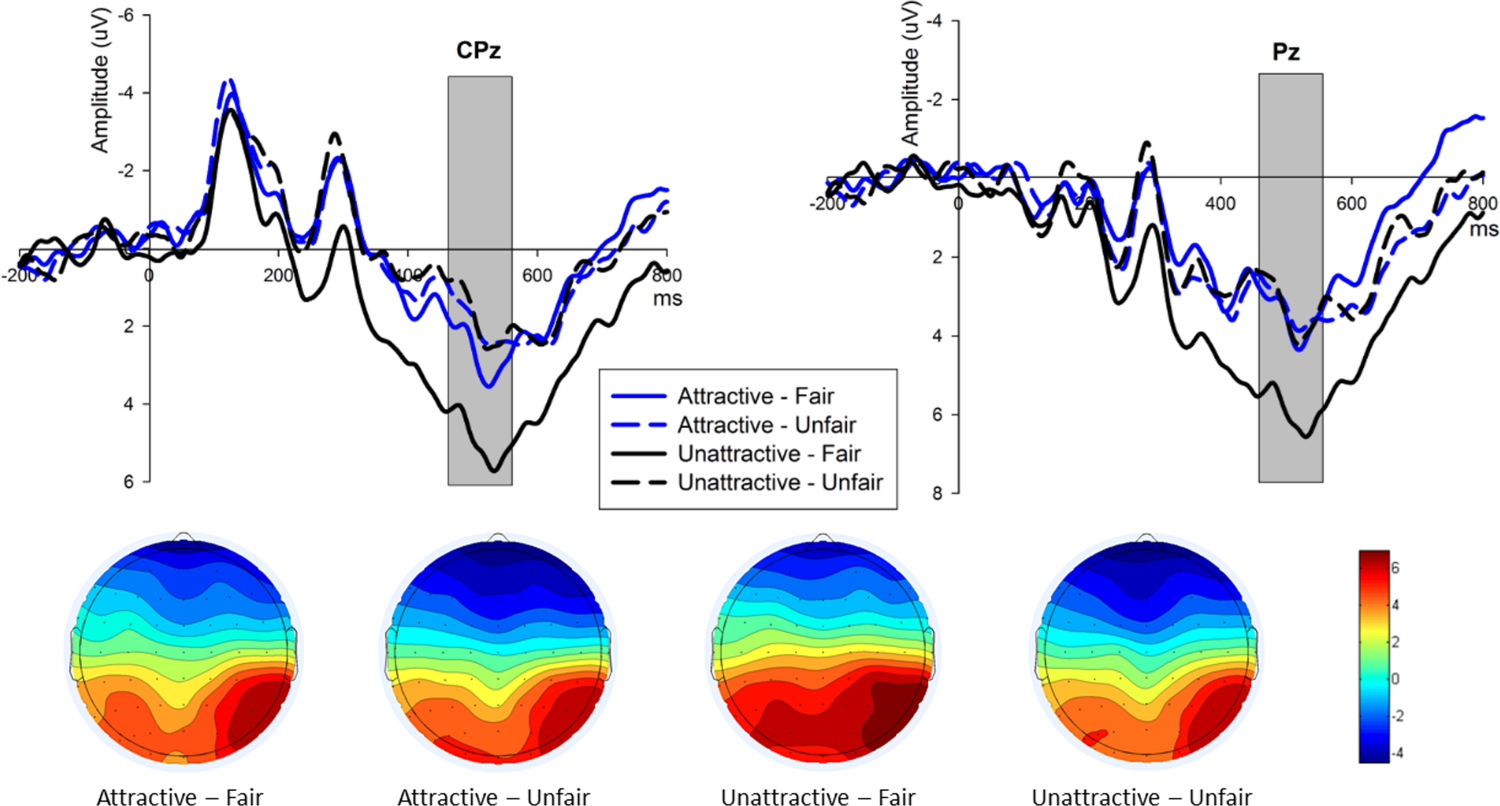
The ERP grand-average waveforms and topographic map of P300. (A) CPz and Pz were selected for P300 (shaded 460–560 ms) waveforms to compare fair and unfair offers in the two face conditions. (B) The bar for four offer conditions of P300 topographic map ranges from -4.5μV to 6.5μV.

Similar to FRN analysis, we excluded mid-offers (3:7) and a two (Attractiveness) × two (offers) × nine (Electrodes) ANOVA for the P300 (460–560 ms post-onset offer presentation) was conducted. A significant main effect for offer was observed as fair offers (Mean ± SE, 5.236 ± 1.390μV) elicited a more pronounced P300 [F_(1,16)_ = 9.656, p = 0.007, partial η^2^ = 0.376] than the unfair ones (Mean ± SE, 3.899 ± 1.267 μV), as depicted in Fig 4. Main effect of attractiveness approached significance [F_(1,16)_ = 3.662, p = 0.074, partial η^2^ = 0.186] between the two face conditions (attractive face: 4.193 ± 1.310 μV, unattractive face: 4.942 ± 1.344 μV). A significant interaction effect [F_(1,16)_ = 7.613, p = 0.014, partial η^2^ = 0.322] between Attractiveness and offer was also observed. In attractive-face condition, similar to the above FRN pattern, no significant effect of Offer were found [F_(1,16)_ = 0.449, p = 0.512, partial η^2^ = 0.027]. In the unattractive-face condition, fair offers (Mean ± SE, 6.072 ± 1.405 μV) exhibited more positive P300 [F_(1,16)_ = 24.105, p < 0.001, partial η^2^ = 0.601] than unfair offers (Mean ± SE, 3.812 ± 1.321 μV).

### 3.6 Mixed model ANOVA

We adopted EEG data of male participants from our previous work [7] and conducted a two (attractiveness) × nine (electrodes) mixed model ANOVA (using gender as between-subjects factor) for both N2 and early frontal LPP. As for N2, a significant main effect of attractiveness [F_(1, 36)_ = 9.701, p = 0.004, partial η^2^ = 0.212] was observed. The interaction effect between Attractiveness and Gender was significant [F_(1, 36)_ = 6.897, p = 0.013, partial η^2^ = 0.161]. The follow up simple effect analysis revealed that unattractive faces (Mean ± SE, 0.730 ± 0.691 μV) elicited more negative amplitudes [F_(1, 20)_ = 13,687, p = 0.001, partial η^2^ = 0.406] than attractive faces (Mean ± SE, 2.088 ± 0.616 μV) for male participants, but were not different for female participants as showed in section 3.3 above. In terms of early frontal LPP, again a significant main effect of attractiveness [F_(1, 36)_ = 16.767, p < 0.001, partial η^2^ = 0.318] was identified, however, the interaction effect between Attractiveness and Gender was not significant [F_(1, 36)_ = 1.001, p = 0.324, partial η^2^ = 0.027]. Attractive faces (Mean ± SE, 2.678 ± 0.752 μV) generated more positive amplitudes [F_(1,20)_ = 14.332, p = 0.001, partial η^2^ = 0.417] than unattractive faces (Mean ± SE, 1.183 ± 0.780 μV) for male participants. Similarly, more positive amplitudes was also elicited by attractive faces compared to unattractive ones for female participants (as evidenced in section 3.3 above).

### 3.7 Questionnaire

The sorting results revealed that the mean importance score of facial attractiveness ranked 5^th^ (Mean = 3.412, SD = 0.795) out of the six factors. Paired t-test showed that Intelligence (t_(16)_ = 3.516, p = 0.003, Cohen’s *d* = 0.85), Healthy body (t_(16)_ = 2.304, p = 0.035, Cohen’s *d* = 0.56) and Achievement (t_(16)_ = 3.392, p = 0.004, Cohen’s *d* = 0.82) were significantly more important than Facial attractiveness. However, they were not different among themselves (Intelligence vs. Healthy body, t_(16)_ = 1.768, p = 0.096, Cohen’s *d* = 0.43); (Intelligence vs. Achievement, t_(16)_ = 0.293, p = 0.773, Cohen’s *d* = 0.07); (Healthy body vs. Achievement, t_(16)_ = 1.167, p = 0.260, Cohen’s *d* = 0.28). Facial attractiveness were not significantly different with Resources (t_(16)_ = 1.102, p = 0.287, Cohen’s *d* = 0.27) and Social status (t_(16)_ = 0.889, p = 0.387, Cohen’s *d* = 0.22).

In order to compare potential gender differences we also surveyed all subjects (total of 21, all males) who participated in our former experiment [7] using the same questionnaire and discovered that facial attractiveness ranked 2^nd^ (Mean = 4.238, SD = 0.831) among the six factors, and it was significantly more important than Resource (t_(20)_ = 4.019, p = 0.001, Cohen’s *d* = 0.88), Social status (t_(20)_ = 3.286, p = 0.004, Cohen’s *d* = 0.72) and Achievement (t_(20)_ = 3.078, p = 0.006, Cohen’s *d* = 0.67), but was not statistically different with Healthy body (t_(20)_ = 1.451, p = 0.162, Cohen’s *d* = 0.32) and Intelligence (t_(20)_ = 1.375, p = 0.184, Cohen’s *d* = 0.30). Moreover, Resource, Social status and Achievement ranked in the bottom three and were not different among each other (Resource vs. Social Status, t_(20)_ = 0.645, p = 0.526, Cohen’s *d* = 0.14); (Resource vs. Achievement, t_(20)_ = 0.568, p = 0.576, Cohen’s *d* = 0.12); (Social Status vs. Achievement, t_(20)_ < 0.001, p > 0.9, Cohen’s *d* < 0.01). Table 2 below illustrates all mean scores and rankings from both questionnaires in relation to male and female subjects.

**Table 2:** The mean score and importance rankings of factors towards opposite sex’s attractiveness.

Female subjects (this experiment)			Male subjects (previous experiment)		
Rank	Mean	SD	Rank	Mean	SD
1. Intelligence	4.412	0.712	1. Healthy body	4.429	0.746
2. Achievement	4.353	0.702	2. Facial Attractiveness	4.238	0.831
3. Healthy body	4.118	0.781	3. Intelligence	3.905	0.700
4. Resource	3.765	0.752	4. Social status	3.381	0.921
5. Facial attractiveness	3.412	0.795	4. Achievement	3.381	0.865
6. Social status	3.177	0.883	6. Resource	3.238	0.944

Furthermore, following the study by Krupp et al [19], we performed mixed factorial analysis of covariance (ANCOVA) for the females’ data (facial attractiveness on acceptance ratio) to control for individual differences in intelligence, achievement, healthy body, resource and social status. We discovered a significant main effect of Attractiveness (F_(1, 16)_ = 11.53, p = 0.006, partial η^2^ = 0.512) and Offer (F_(4, 64)_ = 32.171, p < 0.001, ε = 0.417, partial η^2^ = 0.745). The interaction effect between Attractiveness and Offer was also significant (F_(4, 64)_ = 4.812, p = 0.003, partial η^2^ = 0.304). The results indicated that there is still an effect of attractiveness on acceptance ratio when individual differences are controlled. The simple effect analysis revealed that attractive faces still triggered higher acceptance rates across all five offer (1:9; 2:8; 3:7; 4:6; 5:5) conditions compared with corresponding offers proposed by unattractive-faces (very similar to the behavioral results presented in the acceptance ratio section).

## 4. Discussions

The current experiment evaluated the effect of facial attractiveness on responders’ fairness considerations in a manner similar to our previous study [7] using an “opposite gender experimental setting”. Behavioral results of acceptance ratios were very similar to those typically found in the UG [4, 44]. Participants accepted majority of fair offers, and the acceptance ratio gradually dropped as the offers became less fair. The results also indicated that, female responders had larger tendency to accept all offers (including unfair offers) if they were proposed by an attractive male allocator. It was in line with studies that attractive individuals were often offered more than ordinary-looking individuals [4], and studies that used female as proposers who exhibit “beauty premium” effect to modulate male responders’ fairness consideration [7, 28].

In both attractive and unattractive-face conditions, the RTs of extreme offers (5:5 and 1:9) were significantly different compared to mid-value offer (3:7). This was consistent with the study of Polezzi et al [39] that people consistently make rapid decisions about offers which appear clearly fair or unfair. Mid-value offers (which do not readily fall into one of the two categories) would induce a conflict and require more time for completion of the decisional process. Female subjects responded quicker to fair offer (5:5) and much slower to unfair offer (1:9) from an attractive allocator. More specifically, a clearly visible fluctuation in RTs were observed between fair offer (5:5) and unfair offers (2:8) and (1:9) when an attractive allocator proposed those offers. Subjects rapidly accepted fair offer (5:5) and delayed their decisions for unfair offers (2:8) and (1:9). Intriguingly, when an unattractive allocator proposed similar offers, the RTs appeared to be more stable. This result was in accordance with our previous experiment using opposite gender settings [7] and reinforced the view that attractive allocators would expedite the responders’ decision making process towards a fair offer but also make them hesitated when they face an unfair offer. On the other hand, subjects would follow a predetermined strategy to choose acceptance or rejection at their own pace when facial attractiveness of the allocator is not in salient forms. Preceding studies suggested that strategic decisions in interpersonal context are affected by factors not directly related to monetary benefit [39, 56], and taking together findings from our past researches [7, 28] that facial attractiveness could evoke reward-related neural processing, and was perceived as a rewarding factor for responders, we would conjecture that the “Beauty premium” effect also existed in “male proposer, female responder” UG setting, and it would be universally true that the enjoyment of the “good looks” could potentially attenuate the responders’ dissatisfaction with an unfair money split and making them hesitated to reject unfair offers from attractive allocators.

In contrary to the “female proposer, male responder” UG, the mixed model ANONA analysis (using previous and current ERP data, in the same electrodes and time window) showed different N2 (210–280 ms) and similar early frontal LPP (380–480 ms) effects between attractive-face and unattractive face conditions post-onset face stimulus. Unattractive faces (as compared to attractive ones) elicited more negative N2 amplitudes for males but similar N2 amplitudes were elicited by both attractive and unattractive faces from the frontal area for females (see Fig 2). Our result was supported by the work of Schacht et al [33] that ERPs to attractive and unattractive faces maybe indistinguishable in such an early stage (about 200 ms) post-onset the face presentation and the scalp topographic maps showed these effects were distributed in frontal site. Overall, this study provided further evidence that facial attractiveness might be processed early [33], in line with other studies [7, 22, 23, 57, 58]. However, our results is at variance with other studies that found unattractive faces would induce larger N2, and suggested that men are vulnerable to attractive females and would anticipate to view those females than unattractive ones. When disfavored unattractive faces are presented, they would be in a mental conflict, and in turn, elicit a more negative N2 [7, 27]. Zhang and colleagues [58] identified significant differences of early frontal negativity (around 300 ms post-onset face presentation) when participants made explicit evaluation of attractiveness, and the authors interpreted this early negativity relates to stimulus classification processing, and also might reflect conflict detection during the judgement of faces as to whether or not attractive [58], consistent with other studies [59–61]. As for LPP, mixed model ANOVA results indicated that attractive faces elicit enhanced early frontal LPP than unattractive faces between 380–480 ms time frame, despite of participants’ gender. It is consistent with previous attractiveness investigations and found more positive LPP amplitudes between 350–500 ms for attractive than unattractive faces, suggesting that LPP amplitude is related to the emotional content of a stimulus [32, 62, 63]. In our study, attractive male faces may be more emotionally rewarding to female participants and may then lead to more activation in brain areas of reward processing and emotional arousal [26, 64], consequently generated larger LPP amplitudes.

Although only minimal scalp distribution difference existed when comparing topographic maps of N2 and that of early frontal LPP, in light of the literatures discussed above, we predict these two components observed in the current study might reflect two separate processes or cognitive functions. N2 might reflect attractiveness classification or conflict detection (even though no significant difference was identified) whereas early frontal LPP might reflect an enhanced continued processing of faces or attractiveness evaluation. This notion is also supported by studies which showed that early frontal negativity and late positive component (LPC) in the judgement task could reflect different cognitive functions such as stimulus classification and stimulus evaluation [58, 65]. Furthermore, it is worth noting that the typical LPP amplitudes (between 300–700 ms post-onset stimuli) in parietal electrodes was not different between attractive and unattractive faces. This observation is at variance with previous work conducted by Wiese and colleagues [30] that attractive faces elicit more pronounced LPP in a wide time range (ie., 300–700 ms). The critical differences between our study and this previous study lies in the fact of stimulus selection and task difference in nature. In our study, we adopted only male faces whereas Wiese et al [30] adopted both male and female faces, and hence the facial processing results (after averaging) toward male and female might be different to that toward only males. Also Wiese et al [30] adopted a memory and recognition task, and divided the task into two separate phases (phase 1: gender classification; phase 2: recognition), whereas we adopted a bargaining task (UG) and participants were only asked to view the male photos and follow-up offers to make a decision. However, our LPP pattern was not in line with previous “female allocator, male responder” UG [7]. So what made the discrepancies in both N2 and LPP effects between male and female in relation to face processing? Since the current experiment and the previous opposite-sex UG were identical in methodology, we would infer that there might be cognitive differences across genders in processing facial stimulus. When guys look at girls, they might possess an emphasized sexual appeal [53] in their judgement of female faces or higher anticipation [27] to view more attractive faces so that it might give rise to the attention bias [7, 27, 28] and then might result in an overt cognitive conflict [58, 61] caused by viewing unattractive faces in an early stage (evidenced by larger N2 associated with unattractive faces). In the later stage, they might continue to evaluate facial attractiveness to see which face is more emotionally rewarding [32, 62, 63] to them (as evidenced by distinct early frontal LPP amplitudes elicited between the two faces). Conversely, when girls look at guys, they might be able to classify face types [58] but might not experience the potential attention bias towards unattractive faces and an enhanced facial attractiveness conflict (as evidenced by similar N2 generated by two types of male faces) due to predetermined aesthetic judgement preferences for attractiveness [53]. One possible explanation of this cognitive inconsistency would be due to endogenous gender differences in social psychology. Males would attach more importance to facial attractiveness than their female counterparts when choosing partners, while females tend to primarily consider status and resources as factors ahead of attractiveness [23, 64, 66]. Therefore, the attractiveness level of the opposite sex might have different reward value for men and women [53]. This notion is also supported by Winston et al [24] that men are likely to sacrifice more time, money and effort than women for the opportunity to view attractive opposite-sex faces. Using fMRI, Cloutier and colleagues [53] found that the orbito-frontal cortex (OFC), which was suggested by Dolan [67] as the brain area responsible for evaluating the reward value of ongoing behavior, was activated for males only when viewing attractive female faces. However, apart from the OFC, more areas of the brain’s reward circuit were activated for both males and females when viewing attractive opposite sex’s faces [53]. In the ERP study conducted by van Hooff and colleagues [68], the result suggested that physically attractive or distinctive faces would lead to prioritized processing by both male and females, but the enhanced evaluative processing associated with motivated attention to attractive female faces was found only in males. However, more pronounce early frontal LPP observed in the attractive face condition suggests that being attractive for males still tend to be more emotionally rewarding for females when they begin to evaluate male faces in later stage of cognitive activity, this result well complements the abovementioned studies [4, 21]. It is also worth noting that there was no typical posterior LPP effect observed in the current study which is in contrary with our previous report [7]. It is likely that due to the gender differences discussed above, males might possess a more in-depth continued processing and evaluation of opposite-sex faces than females presumably due to the more motivated attention and emotion attached to view attractive faces.

The follow-up questionnaire indicated that facial attractiveness, being a factor towards attractiveness judgement of individuals, are considered important for both male and female (ratings > 2.5), and hence when it is in place, people would pay much attention towards it. Since facial attractiveness ranked 2^nd^ from the male’s perspective and 5^th^ from the females’ perspective, it may imply that the effect of facial attractiveness on attractiveness judgement of opposite sex might be similar in terms of direction, but might be different in terms of strength as males would perceive facial attractiveness to have greater psychological value than it is for females. Another possible explanation of this ranking results might be due to the structure inequality for women as they are perceived to have lower economic condition and social power [69, 70]. For example, Gibbons [70] carried out a class exercise (Power Game) to test students’ affective and behavioral responses toward unequal distribution of resources. In this experiment, students are divided into groups to make a specified item and the group which complete this task the quickest is the winner. Only one group was given a critical material (a ruler), and not surprisingly, that group became the winner. During discussions after the game, members from the advantageous group reported feelings of achievement, confidence and satisfaction whereas members from disadvantageous groups claimed that they would have won if they had the ruler, and felt discouraged and even depressed. The author also discovered higher rate of depression among female members [70]. The power and status theory of sociology illustrated that negative emotions are mainly the result of low social power [71]. More specifically, in the case of China, females are often in the less-dominated position compared to males [72], so when they look at a male stranger’s face, they may be more inclined to consider much more factors beyond facial attractiveness itself before making (dating or interaction) decisions. In the context of other cultures, Shackelford et al [54] identified 4 dimensions of mate preferences by surveying several thousands of participants around the globe. The results indicated that when choosing mates, women tend to value dependability, stability, education and intelligence whereas men would place more value on good looks, health and desire for home and children [54]. In our study, we didn’t include dependability, stability, education and desire for home and children in the follow up questionnaire analysis, but we did include intelligence, good looks (i.e. facial attractiveness) and health. It’s quite interesting to see that our results don’t seem to deviate much from Shackelford et al [54]’s study. The only difference might be that both women and men would view healthy body and intelligence equally important (no significant differences were found, as identified in section 3.7). However, this difference might be due to the peculiarities of the small sample, but of course, in the current study, our major concern is whether facial attractiveness is ranked differently across male and female participants. After controlling for individual variations, facial attractiveness still had a significant impact on offer acceptance rate. Thus, it is clear (at least in this study) that this impact is likely due to facial attractiveness of male proposers, which is well supported by previous research [7] that there existed a “Beauty Premium” in the two players UG setting. In addition, the questionnaire results may also provide supplementary evidence to further explain why there was a different ‘facial attractiveness effect’ on female subjects compared to their male counterparts in the neuro level during the UG, which well supports our prediction that endogenous gender differences might drive males to attach more motivated attention and emotion to view attractive opposite-sex faces, and hence possess a more in-depth continued processing and evaluation of those faces than when females process male faces. It is also worth noting that although we relied on many trails to increase statistical power during the experiment, this follow-up questionnaire results nevertheless depend on a relative small number of participants. Consequently, any peculiarities of the sample could have an outsized effect on the results. Further research should gather relatively richer samples to test the robustness of the questionnaire findings.

The reinforcement learning theory proposed by Holroyd and Coles [43] indicated that FRN is correlated with the magnitude of the reward prediction error, more specifically, a worse outcome than expected, such as loss or non-reward compared to reward (from the utilitarian feedback perspective). Therefore, in light of this theory, an enlarged FRN would be generated by negative outcomes compared to positive ones, and unexpected outcomes would elicit more negative FRN than expected outcomes. In this study, unfair offers (2:8 and 1:9) generated an enlarged FRN in the frontal-central areas compared to fair ones (5:5 and 4:6). This was in line with existing studies [37, 45]. One possible interpretation of this result was due to an unexpected social norms violation from the subjects’ point of view [6]. We also discovered an interesting discrepancy between attractive-face and unattractive-face conditions when carried out further FRN analysis. In the former condition, both fair and unfair offers elicited similar FRN amplitudes. However in the latter condition, unfair offers elicited more negative FRN amplitudes than fair ones. A similar pattern was observed in the “female allocators, male responder” UG setting [7]. Abundant researches suggested that perceivers judge attractive individuals more favorably than unattractive ones due to the notion of “beauty is good” [73, 74] or the expectation that attractive individuals were judged to be more generous and helpful-looking [13]. Previous fMRI studies examining brain circuitry of reward processing also illustrated that male subjects were willing to spend extra effort to view attractive faces [26], or give away a portion of monetary benefit in exchange for the so called “money equivalent pleasure” by viewing good-looking people [36]. Therefore, it leads us to tender that in the “male allocator, female responder” UG, due to the enjoyment of facial attractiveness, responders would likely surrender certain amount of money for attractive males, and this would affect their subsequent fairness considerations, lead to decreased FRN amplitudes for the negative emotions and the null effect of FRN was resulted eventually. On the other hand, without the potential enjoyment of beauty, the FRN effect was significantly in place, as responders became very sensible towards fair and unfair offers in the unattractive-face condition. This finding is consistent with previous studies [7, 28].

P300 is generally related to the stimuli occurrence process [75]. More specifically, it represents the motivational significance [46] and the allocation of attention during outcome evaluation [76]. In the fair condition of the current study, an increased P300 amplitudes were observed in the parietal scalp sites and was proportional to an increased amount of money offered. This was in line with the notion that P300 amplitude was more pronounced for large than small alternative outcomes and was generally larger for gains compared to losses [46]. Also speaking from the perspective of attention allocation, our result might imply that larger momentary income would capture more attention from the subjects and hence motivate them to value equal divisions more than unequal ones, thus generate more positive P300 responses to fair offers [77, 78]. Furthermore, in the unattractive-face condition, P300 responses for unfair and fair offers followed this general pattern, however, similar to null effect of FRN, again no P300 effect (relative to fair and unfair offers) was identified in the attractive-face condition. This is consistent with our previous experiment with “opposite gender setting” [7], and would be explained by the fact that in the unattractive-face condition, female subjects allocated less attention to unfair offers compared with fair ones, leading to smaller P300 generated by unfair offers. In the attractive condition, the proposers’ facial attractiveness might alleviate the offer effect on P300 so that fair and unfair offers from attractive males captured analogous attention of female subjects. Therefore, due to the lack of differences in FRN and P300 produced by attractive-face conditions with respect to fair and unfair offers, we posit that the “beauty premium” effect would also exist in “male proposer, female responder” context. However, the present study does not provide a baseline condition of “neutral” or mid-attractive faces, and this has been shown to qualify the effects of attractiveness component [19]. It would be interesting for future research to explore whether participants are particularly lenient with attractive faces, particularly critical with unattractive faces, or both. Also, it would be interesting to see FRN and P3 effects for mid-attractive faces.

## 5. Conclusion

In conclusion, the current study delineated the effect of male proposers’ facial attractiveness on female responders’ fairness consideration and their subsequent decision outcome during the UG. Our findings suggest that female responders do appreciate by viewing attractive male proposers’ faces, and would consider attractiveness as a factor to alter their consideration of fairness during the UG. However, due to the insignificant N2 differences between two face conditions and more pronounced early frontal LPP elicited by attractive-face compared to unattractive ones in this study (using females as subjects), along with both significant N2 and early frontal LPP effects identified in the previous experiment (using males as subjects), it might imply that in comparison to males, females might not possess the potential attention bias towards unattractive faces and an enhanced facial attractiveness conflict due to predetermined aesthetic judgement preferences for attractiveness, suggesting that there might be cognitive differences across genders in processing facial stimuli. However, more pronounced early frontal LPP observed in the attractive face condition suggests that being attractive for males still tend to be more emotionally rewarding for females when they begin to evaluate male faces in later stage of cognitive activity. The behavioral results demonstrated higher acceptance ratios to all offers in the attractive-face condition against unattractive-face condition, longer RTs for unfair offers in the attractive-face condition and shorter RTs for unfair offers in the unattractive condition. Fair and unfair offers generated similar FRN and P300 amplitudes only in the attractive-face condition, on this account, we would infer that there might exist a fairness bias in the UG paradigm as subjects’ fairness consideration might be attenuated by allocators’ facial attractiveness.

## Supporting information

S1 Data Zip file containing experimental data. (ZIP)
